# Mild dyserythropoiesis and β-like globin gene expression imbalance due to the loss of histone chaperone ASF1B

**DOI:** 10.1186/s40246-020-00283-3

**Published:** 2020-10-16

**Authors:** Petros Papadopoulos, Athanassia Kafasi, Iris M. De Cuyper, Vilma Barroca, Daniel Lewandowski, Zahra Kadri, Martijn Veldthuis, Jeffrey Berghuis, Nynke Gillemans, Celina María Benavente Cuesta, Frank G. Grosveld, Rob van Zwieten, Sjaak Philipsen, Muriel Vernet, Laura Gutiérrez, George P. Patrinos

**Affiliations:** 1 Department of Cell Biology, Erasmus MC, Rotterdam, The Netherlands; 2 Department of Hematology, Hospital Clínico San Carlos, Instituto de Investigación Sanitaria San Carlos (IdISSC), Madrid, Spain; 3 Department of Blood Cell Research, Sanquin Research and Landsteiner Laboratory, AMC, UvA, Amsterdam, The Netherlands; 4 UMR Stabilité Génétique Cellules Souches et Radiations, Université de Paris and Université de Paris-Saclay, CEA, 18 route du Panorama, 92260 Fontenay-aux-Roses, France; 5 U1274, Inserm, 18 route du Panorama, 92260 Fontenay-aux-Roses, France; 6 Division of Innovative Therapies, UMR1184, Université Paris-Saclay, Inserm, CEA, Center for Immunology of Viral, Auto-immune, Hematological and Bacterial diseases (IMVA-HB/IDMIT), Fontenay-aux-Roses, France; 7 Laboratory of Red Blood Cell Diagnostics, Sanquin Diagnostics, Amsterdam, The Netherlands; 8 Platelet Research Lab -Instituto de Investigación Sanitaria del Principado de Asturias (ISPA)-, Department of Medicine -University of Oviedo-, Oviedo, Spain; 9 Laboratory of Pharmacogenomics and Individualized Therapy, Department of Pharmacy, University of Patras School of Health Sciences, Patras, Greece; 10 Department of Pathology, College of Medicine and Health Sciences and Zayed Center of Health Sciences, United Arab Emirates University, Al-Ain, United Arab Emirates

**Keywords:** Dyserythropoiesis, Hemoglobin switching, Hereditary persistence of fetal hemoglobin (HPFH), Thalassemia, Gene expression, Erythropoiesis, ASF1B, KLF1, BCL11A

## Abstract

The expression of the human β-like globin genes follows a well-orchestrated developmental pattern, undergoing two essential switches, the first one during the first weeks of gestation (ε to γ), and the second one during the perinatal period (γ to β). The γ- to β-globin gene switching mechanism includes suppression of fetal (γ-globin, HbF) and activation of adult (β-globin, HbA) globin gene transcription. In hereditary persistence of fetal hemoglobin (HPFH), the γ-globin suppression mechanism is impaired leaving these individuals with unusual elevated levels of fetal hemoglobin (HbF) in adulthood. Recently, the transcription factors KLF1 and BCL11A have been established as master regulators of the γ- to β-globin switch. Previously, a genomic variant in the *KLF1* gene, identified by linkage analysis performed on twenty-seven members of a Maltese family, was found to be associated with HPFH. However, variation in the levels of HbF among family members, and those from other reported families carrying genetic variants in *KLF1*, suggests additional contributors to globin switching. *ASF1B* was downregulated in the family members with HPFH. Here, we investigate the role of *ASF1B* in γ- to β-globin switching and erythropoiesis in vivo. Mouse-human interspecies ASF1B protein identity is 91.6%. By means of knockdown functional assays in human primary erythroid cultures and analysis of the erythroid lineage in *Asf1b* knockout mice, we provide evidence that ASF1B is a novel contributor to steady-state erythroid differentiation, and while its loss affects the balance of globin expression, it has no major role in hemoglobin switching.

## Background

The expression of the β-like globin genes follows a well-orchestrated developmental pattern, undergoing two essential switches, the first one during the first weeks of gestation (ε to γ), and the second one around birth (γ to β) [1–4]. Therefore, fetal hemoglobin (HbF), composed of two α-globin and two γ-globin chains, is the dominant type of hemoglobin during the fetal stages of development. Around birth, HbF is gradually replaced by adult hemoglobin (HbA), consisting of two α-globin and two β-globin chains. Already at 6 months of age, the major hemoglobin in the circulation is HbA [5]. However, residual amounts of HbF continue to be synthesized throughout adult life, and the amounts vary considerably, with the large majority of adults having less than 1% of HbF. Our understanding of β-like globin transcriptional control is historically based on Mendelian models of inheritance of natural mutants. Indeed, a series of genetic variants of the β-globin cluster have been discovered that impair the fetal-to-adult hemoglobin switch, leading to persistent γ-globin expression and elevated HbF throughout adult life. This condition is termed hereditary persistence of fetal hemoglobin (HPFH) [6]. There are two types of HPFH variants in the β-globin locus: point mutations in the promoter of the γ-globin genes (*HBG1* or *HBG2*) and deletions removing substantial regions of the β-globin cluster, often including the β-globin gene (*HBB*) [7]. A range of conditions characterized by HbF levels that do not fit clear Mendelian inheritance models or the typical HPFH phenotype, i.e., high HbF levels accompanied by concomitant lower HbA_2_ (α_2_δ_2_) levels, have been previously reported. Although some of this variability can be explained by the β-globin cluster haplotype, a substantial proportion of the HbF increase is not linked to the β-globin cluster, suggesting multi-factorial genetic players in the fine-tuning of β-like globin gene expression regulation.

It is now evident that common HbF variation is a quantitative genetic trait, shaped by common polymorphisms in genes that are not related to the human β-like globin gene cluster. There are only a few examples of such SNPs in genes, affecting for example *BCL11A* (MIM 606557) residing on chromosome 2 [8–11] and the intergenic region between the *MYB* (MIM 189990) [12] and *HBS1L* (MIM 612450) genes that resides on chromosome 6q22.3 [7, 13–15]. These genes have been identified by genome-wide association or traditional linkage analysis approaches. In addition, it has been previously reported that genetic variants in the *KLF1* (MIM 600599) gene are causative for HPFH, in part by rendering the expression of *BCL11A* low [16–18]. The identification of some of the direct transcriptional regulatory regions within the β-like globin gene cluster targeted by these transcription factors has been reported [15, 19–21].

The extremely high variation of HbF levels observed in carriers of the *KLF1* p.K288X variant led us to further examine other potential contributors to the fine-tuning of the γ- to β-globin gene expression balance [22].

Whole transcriptome analysis performed on mRNA from primary erythroid cultures revealed that expression levels of *ASF1B* (MIM 609190), located in chromosome 19, close to the region that showed the highest LOD scores when using multi-point parametric analysis at a penetrance of 90% [16], were downregulated to 70% in HPFH family members. *ASF1B*, anti-silencing function 1B histone chaperone gene, is a member of the evolutionary conserved family of H3/H4 histone chaperone proteins. In humans and mice, there are two paralogues of the Asf1 protein in yeast, namely *ASF1A* (MIM 609189) and *ASF1B* [23–26]. Both ASF1A and ASF1B participate in DNA synthesis-independent assembly of histone H3.3 into chromatin [27]. However, *ASF1A* and *ASF1B* are distinct in expression pattern and function. While *ASF1A* is ubiquitously expressed, *ASF1B* is expressed in highly proliferating tissues, and its expression levels decrease in terminally differentiated and quiescent cells [28]. It was described previously that ASF1B and the histone H3.3 promote pancreatic β-cell proliferation in a synergistic manner [29]. The ASF1B protein is the substrate of the tousled-like kinase family of cell cycle-regulated kinases [30–32] and is thought to play a key role in modulating the nucleosome structure of chromatin by ensuring a constant supply of histones at sites of nucleosome assembly [27], while interacting directly with transcription regulators [23, 24, 33, 34]. ASF1B also cooperates with chromatin assembly factor 1 (CAF-1) to promote replication-dependent chromatin assembly [35, 36], but it does not participate in replication-independent nucleosome deposition mediated by ASF1A and HIRA [28, 37]. Mouse-human interspecies ASF1B protein identity is 91.6%.

In the present study, we have employed lentivirus-mediated knockdown in primary human erythroblast cultures and analyzed the *Asf1b* KO mice [38] to demonstrate that ASF1B histone chaperone is functionally linked to steady-state erythropoiesis. Our data shows that loss of ASF1B compromises steady-state erythropoiesis with modest effects on the regulation of the β-like globin genes.

## Results

### ASF1B is downregulated in human primary erythroid progenitors carrying the p.K288X KLF1 variant

A SNP in the *KLF1* gene resulting in p.K288X protein variant, causative of the inherited HPFH in 10 out of 27 recruited members of a Maltese family, was reported previously [16]. The identification of the point mutation in the Maltese family was achieved through SNP linkage analysis, which pointed to a region in chromosome 19p13.12-13 that showed the highest LOD scores when using multi-point parametric analysis at a penetrance of 90% [16]. No evidence of linkage was observed to other regions, including the *HBB* locus and the HPFH-linked loci of chromosomes 2q33 and 6q22.3. However, HbF levels ranged from ~ 3% to almost 20%, suggesting other contributing factors to the variation of HbF levels among HPFH family members [16].

**Fig. 1:**
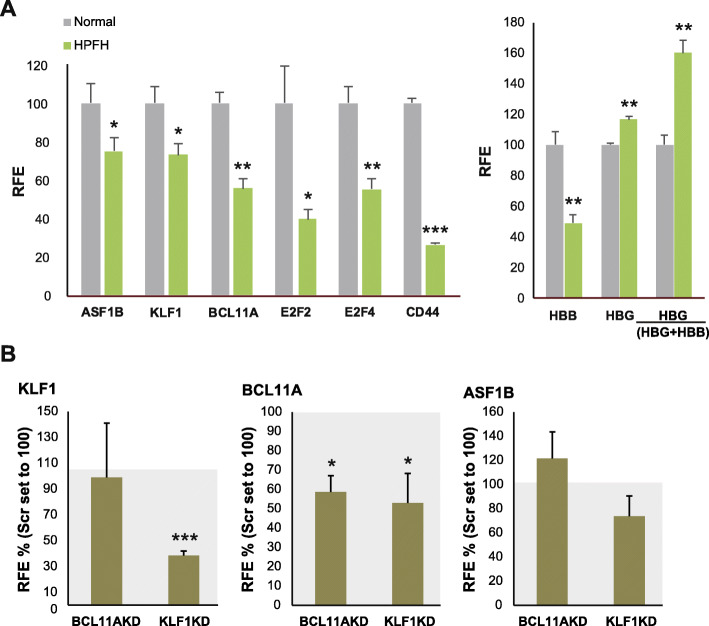
ASF1B is downregulated in the primary erythroid progenitors carrying the p.K288X KLF1 variant. **a** Expression analysis from publicly available microarray data (GSE22109) [16] derived from cultured primary erythroid cells (HEPs) of Maltese family members with HPFH (green bars, *n* = 3) and normal family members (gray bars, *n* = 3). Family members with HPFH display reduction of BCL11A, ASF1B, E2F2, E2F4, CD44, and HBB and increased HBG/(HBB+HBG) ratio. Mean and standard deviation are depicted. *T* test *p* values are indicated. *p < 0.05; **p < 0.005; ***p < 0.0005. **b** Analysis of KLF1, BCL11A, and ASF1B expression by RT-qPCR on HEPs derived from buffy coats from healthy individuals and transduced with shRNA lentiviruses targeting KLF1 and BCL11A. Knockdown of either factor does not influence significantly the ASF1B expression. Expression levels are normalized setting expression levels of mock knockdown controls at 100. Mean and standard deviation are depicted. Shadowed box at 100 corresponds to the expression levels in mock knockdown controls. *T* test *p* values are indicated. *p < 0.05; **p < 0.005; ***p < 0.0005

Examining in more detail the region in chromosome 19 that showed the highest LOD scores and the expression in erythroid primary cultures of the neighboring genes in close proximity (GSE22109), we identified *ASF1B*, which was found to be downregulated in HPFH family members (Fig. 1a) [16]. As shown in Fig. 1a, and as previously reported, KLF1 targets *BCL11A*, *CD44* (MIM 107269), *E2F2* (MIM 600426), *E2F4* (MIM 600659), and *HBB* (MIM 141900), were significantly downregulated, and the γ-globin versus total γ+β-globin expression ratio was increased in primary erythroid cells derived from HPFH individuals [16]. We confirmed that *ASF1B* mRNA levels were significantly reduced to 70% in HPFH family members presenting with the highest HbF levels. Since we could not exclude that the transcription of *ASF1B* is dependent on KLF1 or BCL11A activities, we performed knockdown of either KLF1 or BCL11A in healthy human primary erythroid progenitors and analyzed the *ASF1B* expression levels accordingly. *BCL11A* downregulation did not affect the expression of *ASF1B* at all, while KLF1 knockdown resulted in a small reduction of *ASF1B* expression which did not reach statistical significance (Fig. 1b). This suggests that the significant reduction of *ASF1B* expression levels in primary erythroid cells cultured from the HPFH family members might be partially, but not exclusively, due to transcription regulation by KLF1.

### Knockdown of ASF1B in human primary erythroid cells induces γ-globin expression

**Fig. 2:**
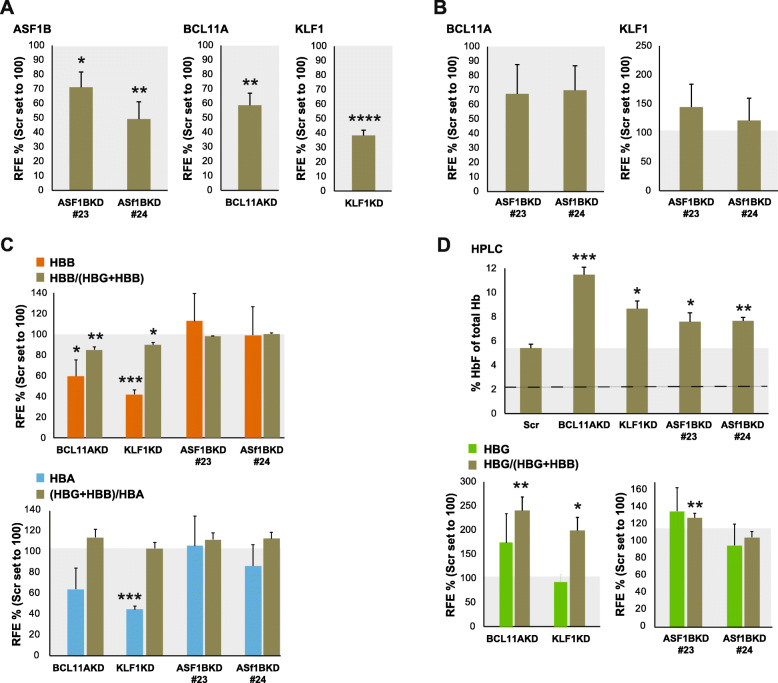
Knockdown of ASF1B in human primary erythroid cells induces γ-globin expression. **a** Expression analysis by RT-qPCR of ASF1B, BCL11A, and KLF1 (last two taken from Fig. 1b) on shRNA lentivirus-transduced HEP cultures. Expression levels are normalized setting expression levels of mock knockdown controls (Scr) at 100. Mean and standard deviation are depicted. Shadowed box at 100 corresponds to the expression levels in mock knockdown controls. *T* test *p* values are indicated. **b** The expression of BCL11A and KLF1 is not significantly affected in ASF1B knockdown HEPs as analyzed by RT-qPCR. Expression levels are normalized setting expression levels of mock knockdown controls (Scr) at 100. Mean and standard deviation are depicted. Shadowed box at 100 corresponds to the expression levels in mock knockdown controls. *T* test *p* values are indicated. **c** *HBB* and *HBA* expression analysis by RT-qPCR. Knockdown of either KLF1 or BCL11A has an effect on *HBB* expression, whereas ASF1B knockdown does not, while overall, *HBA* expression is not affected. Expression levels are normalized setting expression levels of mock knockdown controls (Scr) at 100. Mean and standard deviation are depicted. Shadowed box at 100 corresponds to the expression levels in mock knockdown controls. *T* test *p* values are indicated. **d** RT-qPCR of *HBG* expression and HPLC analysis of hemoglobin composition show the increase in HbF after knockdown of ASF1B in HEPs. Values are normalized to those measured in mock knockdown controls (Scr). Mean and standard deviation are depicted. Shadowed box at 100 corresponds to the expression levels in mock knockdown controls. *T* test *p* values are indicated

To functionally assess the consequences of *ASF1B* downregulation on human β-globin gene expression, we performed knockdown experiments on primary human erythroblasts derived from healthy donors. We used three shRNA lentiviral constructs against *ASF1B*, of which two (#23 and #24, Supplementary Table 1) resulted in efficient knockdown as measured by RT-qPCR. Therefore, we used those two for further analysis. We included *BCL11A* and *KLF1* lentiviral shRNA-transduced cells as the positive controls for γ-globin reactivation and a non-specific scrambled (Scr) shRNA as the negative control. After lentiviral transduction and 24h selection with puromycin, cells were induced to differentiate (+Epo and holo-transferrin), and after 24 h, cells were collected for analysis. We confirmed the shRNA-mediated downregulation of *BCL11A*, *KLF1*, and *ASF1B* transcripts by RT-qPCR (Fig. 2a). We then investigated the impact of *ASF1B* knockdown on BCL11A and KLF1 expression. We found that knockdown of *ASF1B* did not affect the expression of KLF1 and BCL11A levels significantly (Fig. 2b). It is known that one of the principal targets of KLF1 is the *HBB* gene (encoding β-globin) [39, 40], and we could verify that by knocking down the *KLF1* gene expression (Fig. 2c). Indirect downregulation of *HBB* gene transcription, due to the upregulation of γ-globin expression, has been reported upon BCL11A knockdown in human proerythroblasts [41]. We also observed this in our human erythroid cultures (Fig. 2d). In contrast, *ASF1B* knockdown did not reduce the levels of *HBB* mRNA expression (Fig. 2c). *HBA* expression levels were also not altered significantly in the ASF1B KD experiments (Fig. 2c). When measuring hemoglobin composition from the lentiviral-transduced HEPs by qRT-PCR and HPLC, we observed that ASF1B knockdown increased γ-globin expression, as shown by the percentage of HbF of total hemoglobin in the samples (Fig. 2d).

### Asf1b KO mice display mild dyserythropoiesis

**Fig. 3:**
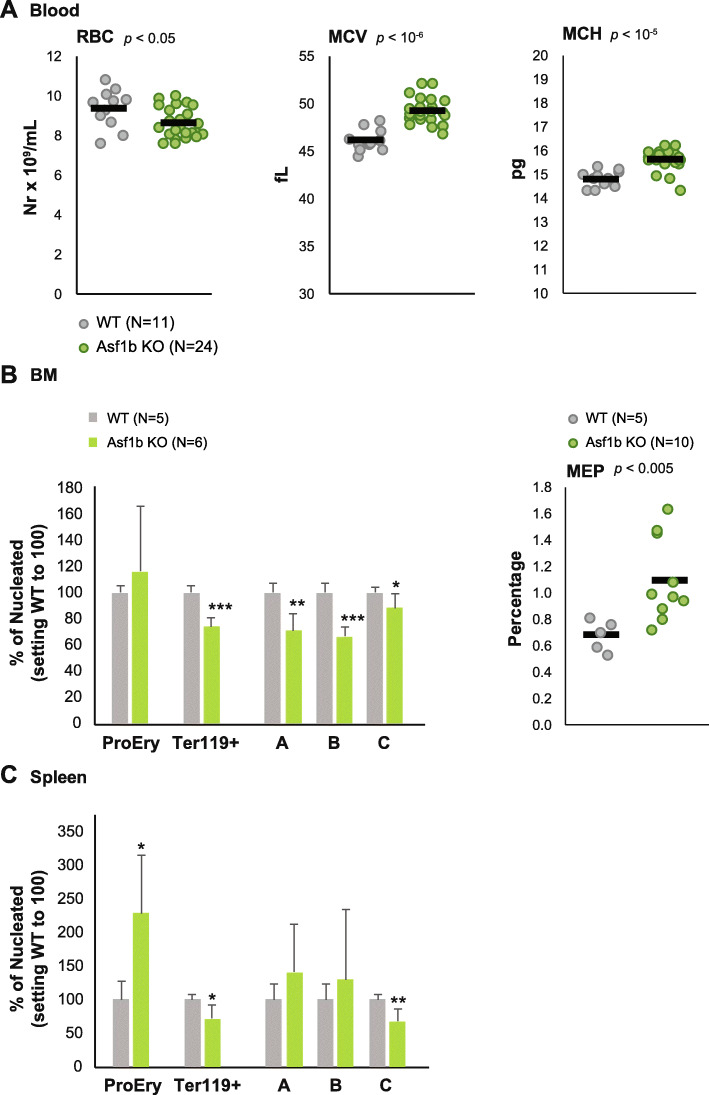
*Asf1b* KO mice display mild dyserythropoiesis. **a** Complete blood count of blood samples from *Asf1b* KO mice and WT littermates. The red blood cell (RBC) counts, the mean corpuscular volume (MCV), and mean corpuscular hemoglobin (MCH) are depicted on individual animals. The black bar is the mean. *T* test *p* value is indicated. **b** Left, bar graph depicting the flow cytometry analysis of the erythroid compartment in the bone marrow of *Asf1b* KO and WT littermates following the gating strategy of Socolovsky [42]. Right, dot plot depicting the basal percentage of megakaryocyte-erythroid progenitors in the bone marrow of *Asf1B* KO mice and WT littermates. The black bar is the mean. *T* test *p* value is indicated. **c** Bar graph depicting the flow cytometry analysis of the erythroid compartment in the spleen of *Asf1b* KO and WT littermates following the gating strategy of Socolovsky [42]

To investigate the potential contribution of ASF1B in erythropoiesis and globin gene expression regulation, we set out to study these processes in *Asf1b* knockout (KO) mice. Complete blood count was performed on blood samples from adult *Asf1b* KO mice and wild-type (WT) littermates (Fig. 3a). Interestingly, *Asf1b* KO mice present with a reduced red blood cell (RBC) count (8.62 versus 9.34 × 10^9^ cells/ml; *p* < 0.05), an increased mean cell volume (MCV 49.15 versus 46.10 fL; *p* < 10^7^), and an increased mean corpuscular hemoglobin (MCH 15.62 versus 14.80; *p* > 10^−6^), suggestive of compensatory anemia. Their spleen was significantly enlarged (116.91 versus 90.20 mg; *p* < 0.05), supporting this notion, considering the increased tendency of the body size of *Asf1b* KO mice that did not reach statistical significance (Table 1).

**Table 1:** Body and spleen weight of *Asf1b* KO mice and WT littermates. Mice included in this analysis are female littermates from heterozygous crossings. The body weight, spleen weight, and spleen weight/body weight ratio are shown. The mean and standard deviation are indicated. *T* test *p* values are given

Genotype	Body weight (g)	Spleen weight (mg)	Spleen/body weight
WT (*N* = 10)	25.93 ± 2.67	90.20 ± 13.54	3.50 ± 0.54
*Asf1b* KO (*N* = 8)	28.38 ± 4.26	124.13 ± 28.09	4.56 ± 1.77
*p* value	NS	< 0.05	NS

*NS* not significant

We next set out to study the erythroid compartment in the bone marrow and spleen of *Asf1b* KO mice (Fig. 3b, c). The bone marrow presented an accumulation of early erythroid progenitors (CD71^+^Ter119^neg^) and a significant reduction of the Ter119 positive cells, “A” (CD71^+^FSC^high^), “B” (CD71^+^FSC^low^), and “C” (CD71^neg^FSC^low^) corresponding to more mature erythroid cells (Fig. 3b, bar graph), based on the gating strategy by Socolovsky [42]. When the megakaryocyte-erythroid-progenitor (MEP) fraction was analyzed, we observed an increase of these progenitors in *Asf1b* KO bone marrow (Fig. 3b, dot plot). Analysis of the splenic erythroid compartment revealed a significant increase of early erythroid progenitors (proerythroblasts; CD71^+^Ter119^neg^), accompanied by a significant reduction of the more mature cells, specially the population “C,” indicative of moderate stress erythropoiesis (Fig. 3c). Altogether, these data suggest that *Asf1b* KO mice present with mild dyserythropoiesis and have engaged stress erythropoiesis in the spleen.

### Persistence of embryonic globin expression in adult Asf1b KO mice

**Fig. 4:**
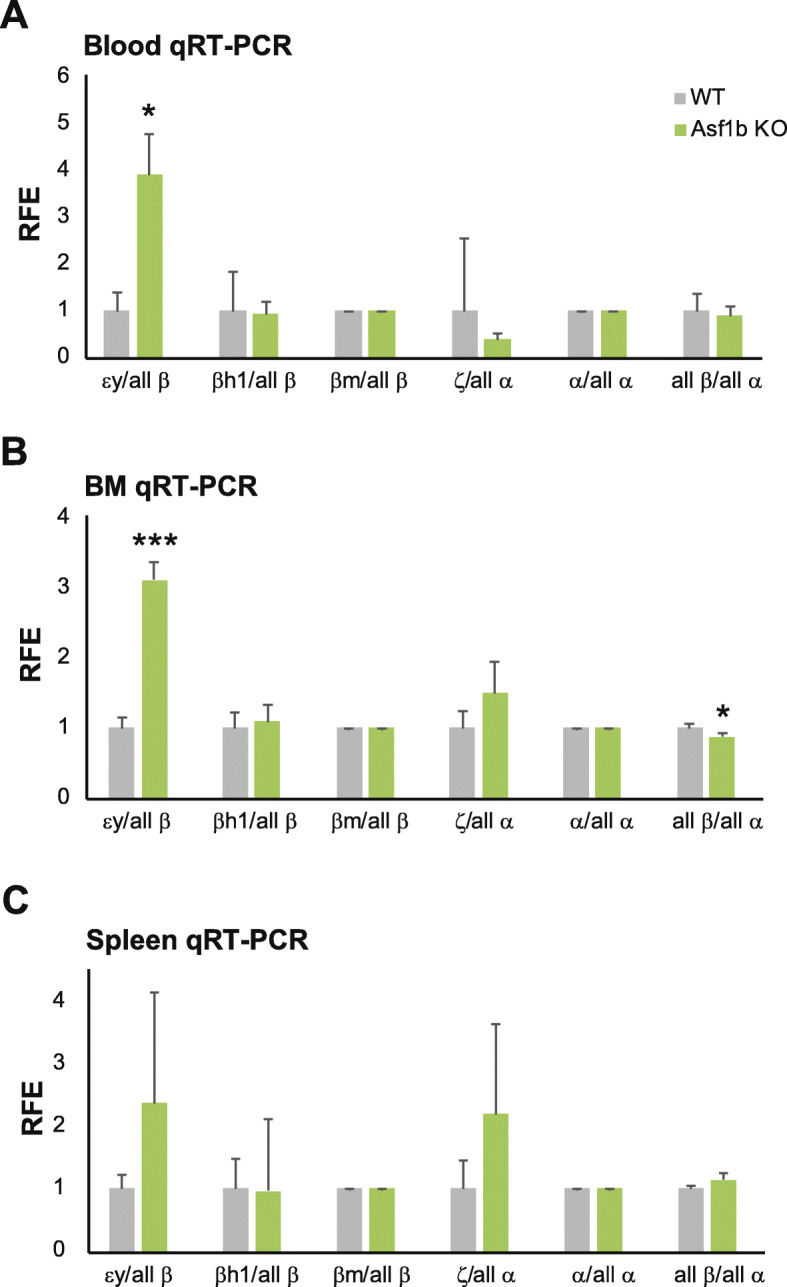
Persistence of embryonic globin expression in adult Asf1b KO mice. Expression analysis by RT-qPCR of β-like and α-like globin genes in the blood (**a**), bone marrow (**b**), and spleen (**c**) samples from *Asf1b* KO mice and WT littermates. Ratios of relative fold enrichment (RFE) of a given β-like or α-like globin versus total β- or α-globin, respectively, are depicted. Mean and standard deviation are represented, and *T* test *p* values are indicated. *p < 0.05; ***p < 0.0005

We next set out to analyze the expression of the β-like globin genes in the blood, bone marrow, and spleen of *Asf1b* KO mice and WT littermates. In mice, the developmental pattern of β-like globin gene expression is marked by the expression of εy and βh1 in the embryonic stage, which is replaced by βmaj + βmin at the fetal stage. RT-qPCR revealed significant upregulation of the expression of εy-globin in the blood and bone marrow, with a modest upregulation in the spleen which did not reach statistical significance (Fig. 4). The upregulation of εy-globin hemoglobin occurred without major alterations in the expression levels of other embryonic globins, i.e., βh1- and ζ-globin. A significant decrease, although very mild, was identified in the bone marrow in the total amount of β-globin expression compared to the total amount of α-globin (Fig. 4b). This imbalance was not detected in the blood of *Asf1b* KO mice (Fig. 4a). This suggests that the normal α/β-globin ratio is restored via a post-transcriptional mechanism, reminiscent of erythropoiesis in individuals with thalassemia trait. Thereof, we conclude that the increased expression of εy-globin is not due to hampered expression of the other globins. Collectively, these data indicate that although not to the same extent as it occurs when knocking down *BCL11A* (direct regulator) or *KLF1* (indirect regulator via *BCL11A* and *HBB*), lower expression levels of *ASF1B* promote γ-globin expression, apparently without reducing *HBB* gene expression. Our data show that *ASF1B* loss of function in mice causes mild dyserythropoiesis with the persistence of εy globin in adult hematopoietic tissues, and thus supporting the notion that the imbalance of β-like globin expression might be due to dysregulated erythroid differentiation, rather than to a direct role of *ASF1B* in globin switching.

## Discussion

In this study, we report on the role of ASF1B in erythropoiesis and globin gene expression.

ASF1B is known to function as a histone chaperone protein important in chromatin remodeling [27] and nucleosome assembly and disassembly, and when knocked down, it is also known to influence gene expression [43]. It has been recently reported that ASF1B might play a role in epigenetic reprogramming [44, 45], and furthermore, in alternative telomere lengthening [46]. However, the complete spectrum of ASF1B functions has not yet been asserted, and especially interesting is the reported cooperation between ASF1B and general transcription factors such as the TFIID [24, 33]. Microarray analysis on erythroid cells from a family carrying KLF1 p.K288X variant leading to HPFH showed that family members with high HbF levels had reduced *ASF1B* gene expression levels compared to family members with normal (low) HbF levels. We studied the possibility of KLF1 directly regulating *ASF1B* expression by performing knockdown experiments on primary HEPs. However, we did not observe significant downregulation of *ASF1B* upon *KLF1* knockdown, possibly due to the short-term nature of the knockdown assays. It has been reported that *ASF1B* gene expression is regulated by E2F factors [47, 48]. Since *E2F2* and *E2F4* are direct targets of KLF1, and also downregulated in HPFH family members (Fig. 1a), we cannot exclude an indirect contribution of the *KLF1* variant to the downregulation of ASF1B expression via *E2F2/E2F4*.

Importantly, the microarray data shows a significant decrease of *HBB* gene transcription in those family members with high HbF levels, similarly to what has been documented for *cis*-acting genetic variants or SNPs in the *HBB* gene promoter CACCC box (see also http://globin.bx.psu.edu/hbvar) [20, 21] to which KLF1 binds. In addition, γ-globin gene expression is upregulated. Thus, it is the balance β- versus γ-globin expression that results in the high percentage of HbF, in this case, by directly affecting the expression of γ- and β-globin in an opposite manner.

Since microarrays are not suitable for measuring globin expression due to saturation of the probe sets, we confirmed these dynamics with RT-qPCR analysis in knockdown assays. The results of knockdown experiments of KLF1 and BCL11A in adult erythroid progenitors from healthy donors agreed with such an observation, i.e., increase of HbF levels compared to the control shRNA, with accompanying decrease in β-globin expression (Fig. 2). This effect on β-globin expression downregulation has been seen in single and compound knockout mouse models for *Klf1* and *Bcl11a* [49]. Interestingly, knockdown of ASF1B resulted in an increase of HbF, without significantly altering KLF1, BCL11A, or β-globin expression (Fig. 2).

It has been recently reported that KLF1 recruits histone chaperones HIRA and ASF1A to maintain an open β-globin locus and that this recruitment is necessary for proper β-globin transcription [40]. Although ASF1A and ASF1B are paralogues, it is known that ASF1B does not cooperate with the ASF1A/HIRA complex and that it performs separate actions on nucleosome assembly [28, 37]. Our results are therefore in agreement with this notion, since depletion of ASF1B did not affect β-globin expression, neither in the *Asf1b* KO mouse model, nor in human erythroid progenitors where ASF1B had been knocked down.

The analogy between KLF1 and ASF1B goes further regarding general erythropoiesis. It has been recognized that a special case of a genetic variant of KLF1 is causative of congenital dyserythropoietic anemia (CDA) variant (type IV) in addition to the accompanying HPFH already reported. Interestingly, the two genes involved in the development of CDA type I are CDAN1 and C15ORF41, both of which have been reported to physically interact with ASF1B [50–55]. This suggests that shared functions by the interacting proteins ASF1B, CDAN1, and C15ORF41 might be crucial during erythropoiesis and for β-like globin expression regulation.

Indeed, loss of function studies in *Asf1b* KO mice revealed mild dyserythropoiesis and persistence of εy-globin expression in adult hematopoietic tissues, without altering the expression levels of other β-like globins. While there was a statistically significant reduction of the total β-like globins compared to α-globins, this was only identified in the bone marrow, and it was very mild.

These results, which are in total concordance with our observations in human cellular models of erythropoiesis, indicate that ASF1B fine-tunes erythropoiesis in vivo.

It has been suggested that ASF1B deficiency results in cell cycle defects due to impaired nucleosome assembly [26, 35, 36, 54, 56]. In this regard, a defective shorter cell cycle during erythropoiesis might result in the production of immature, “skipping division” erythroid cells, which contain higher levels of HbF. In fact, hemolytic treatment in mice or baboons results in an increase of erythroid cells containing fetal/embryonic globin [57, 58]. It has also been described that ASF1B interacts with TFIID [24]. Such interactions involving also transcription regulators, which we have previously shown to be important for mouse erythropoiesis [59], could have a major impact in the coordination between chromatin remodeling, chromatin accessibility, and transcription regulation, and affect consequently gene expression of the β-like globin genes [24, 33]. Experimental evidence of how ASF1B is involved in these processes (cell cycle/replication, transcription, epigenetic regulation) and its role in the molecular mechanism behind the transcription/silencing of the γ-globin genes is missing from our work. Identification of interactors of ASF1B by immunoprecipitation and mass spectrometry as well as cell cycle analysis in erythroid cells should contribute in this direction and should be the subject of future studies.

## Conclusions

In essence, our data indicate that ASF1B cooperates to adult steady-state erythroid differentiation and, most probably associated with this mechanism, it has an accessory function in the silencing of the embryonic globin genes. At present, the exact mechanism by which ASF1B exerts its function related to globin gene expression remains unclear, and further experiments should focus on the molecular characterization of the protein.

## Methods

### Mice

Asf1b knockout mice were generated as described previously [38]. All animal studies were performed in compliance with the European Community regulation for laboratory animal care and use (Directive 2010/63/UE). The mouse facility of the CEA at Fontenay-aux-Roses received the agreement delivered by the French Ministry of Agriculture (no. D92032-02). Mice were sacrificed by cervical dislocation. All efforts were made to minimize animal stress and suffering. Mice were housed in controlled 12-h light:12-h dark conditions (lights on from 08:00 to 20:00 h) and were supplied with commercial food and tap water ad libitum. The colony of mice was maintained by breeding heterozygous mice, so knockout mice could be compared with wild-type littermates and all mating pairs in the same mouse facility. Mouse samples were collected for analysis at 3–8 months of age.

### Flow cytometry

Bone marrow and spleen single-cell suspensions were labeled with respective antibody cocktails. For the analysis of the erythroid compartment, CD71-APCCy7 and Ter119-Pacific Blue antibodies were used (BD), and successive stages of erythroid maturation were calculated as previously described [42]. For MEP analyses, the BM cells were first treated with a 0.75% NH_4_Cl solution (Sigma-Aldrich, St. Louis, MO, USA) to lyse red blood cells and labeled with a biotinylated lineage cell detection cocktail (Miltenyi Biotec) and revealed by streptavidineFluor450 (eBioscience). The cells were then labeled with PE-conjugated anti-CD34, APCCy7-conjugated anti-c-Kit, APC-conjugated anti-Sca-1, and PECy7-conjugated anti-CD16/32 (eBioscience) antibodies, and MEPs were identified as Lin−, Sca-1−, c-Kit+, CD16/32−, and CD34− [60]. At least 300,000 cells were acquired.

The cells were analyzed using a FACSCanto II and a SORP LSRII (BD) equipped with blue (488 nm), violet (405 nm), and red (638 nm) lasers. Data were analyzed with the FlowJo software.

### Cell cultures

Human erythroid progenitor cells (HEPs) were cultured as described [16, 61] in the presence of recombinant human Epo (Eprex, 1 U/ml), recombinant human SCF (100 ng/ml), and dexamethasone (10^−6^ M, Sigma) from processed buffy coats of active blood donors (Sanquin Blood Bank), according to the guidelines of the Declaration of Helsinki and after approval of the local ethical committee. Cells were cultured at 1.5–3 × 10^6^/ml through daily dilutions or medium replacement. Cells were counted with an electronic cell counter (CASY-1, Schärfe-System, Germany). To induce terminal differentiation, cells were washed and transferred to medium with recombinant human Epo (Eprex, 10 U/ml) and a high concentration of iron-loaded transferrin (Sigma, 0.5 mg/ml). Cells were harvested for further analysis (RNA extraction and HPLC) after 24 h in the differentiation medium.

### Transduction of human proerythroblasts with shRNA lentiviral constructs

Lentivirus was produced by transient transfection of HEK 293T cells according to the standard protocols [16, 62]. Two days after transfection, the supernatant was collected, filtered, and concentrated by centrifugation at 20k rpm for 2 h at 4 °C. HEPs cultured for 1 week from fresh buffy coats from healthy volunteers (Sanquin Blood Supply) were transduced in 24-well plates. We used 0.5 × 10^6^ cells per well and sufficient amounts of virus to transduce ~ 80% of the cells at day 1. Puromycin (1 μg/ml final concentration) was added to the cells at day 2, and the selection was performed for 24 h. At day 3, cells were induced to differentiate and were harvested at day 4 for RNA extraction and HPLC analysis as described above. The clones from The RNAi Consortium (TRC [63]; Sigma) used were as follows: the non-target SHC002 vector (Scrambled “Scr” control; SHC002: 5′-CAACAAGATGAAGAGCACCAA-3′), KLF1 shRNA clone TRCN0000016276, [16] BCL11A shRNA clone TRCN0000033449 (validated by us), and ASF1B shRNA clones TRCN0000074225, TRCN0000074226, and TRCN0000074227, of which only the last two gave significant knockdown. Sequences are listed in Supplementary Table 1.

### High-performance liquid chromatography

Separation and quantification of Hb fractions were performed by high-performance cation-exchange liquid chromatography (CE-HPLC) on Waters Alliance 2690 equipment (Waters, Milford, MA, USA). The protocol consisted of a 30-min elution over a combined 20–200 mM NaCl and pH 7.0–6.6 gradient in 20 mM BisTris/HCl, 2 mM KCN. The column, a PolyCAT A 100/4.6-mm, 3-μm, 1500-Å column, was purchased from PolyLC (Columbia, MD, USA) [64].

### RNA extraction and RT-qPCR analysis

Total RNA (1 μg) from each harvested erythroid cell sample after extraction by TRIzol (Invitrogen) was converted to cDNA with SuperScript II reverse transcriptase according to the manufacturer’s instructions (Invitrogen, Paisley, UK). For mouse tissues, the RNeasy Mini Kit (Qiagen) was used to isolate total RNA according to the manufacturer’s instructions. Primers used to amplify *BCL11A*, *ASF1B*, *E2F2*, *E2F4*, *HBB*, and *HBG1/HBG*2 human genes and mouse globin genes plus controls are listed in Supplementary Table 1.

All amplifications took place with SYBR Green PCR Master Mix (Applied Biosystems). RT-qPCR was performed on the Bio-Rad Optical IQ Thermal Cycler (Bio-Rad) under the following conditions: 50 °C for 2 min and 95 °C for 10 min, followed by 45 cycles of 95 °C for 30 s and 62 °C for 30 s. All reactions were performed in triplicate.

Target gene expression was normalized to GAPDH expression. Gene expression levels were calculated with the 2(−DeltaDeltaC(T)) method [65].

## Supplementary information

**Additional file 1.** List of shRNA clones and primer sets used.

## Data Availability

Data sharing is not applicable to this article as no datasets were generated or analyzed during the current study.
