# Supplementary material for: Mild dyserythropoiesis and β-like globin gene expression imbalance due to the loss of histone chaperone ASF1B
Source: Hum Genomics. 2020 Oct 16;14:39. doi: 10.1186/s40246-020-00283-3 (PMC7566067; doi:10.1186/s40246-020-00283-3)
Supplement: Supplementary file 1 [file 40246_2020_283_MOESM1_ESM.docx]

Name	Sequence	Purpose
TRCN0000016276	GCAGAGGATCCAGGTGTGATA	shRNA KLF1
TRCN0000033449	CGCACAGAACACTCATGGATT	shRNA BCL11A
TRCN0000074225	GAGTGGAAGATCATTTATGTT	shRNA ASF1B #22
TRCN0000074226	CCCACTCAACTGCACTCCTAT	shRNA ASF1B #23
TRCN0000074227	AGTGGGCTACTACGTCAACAA	shRNA ASF1B #24
Hs ASF1B Forward	GGTTCGAGATCAGCTTCGAG	qRT-PCR
Hs ASF1B Reverse	ACCAGCACCGAGTCTAGGAT	qRT-PCR
Hs BCL11A Forward	GCCCCAAACAGGAACACATA	qRT-PCR
Hs BCL11A Reverse	TTGCAAGAGAAACCATGCAC	qRT-PCR
Hs E2F2 Forward	AGCTGGAACCGAGAGAACATG	qRT-PCR
Hs E2F2 Reverse	ACACGACCAGGCGAAACC	qRT-PCR
Hs E2F4 Forward	GGGCAGAAGAAGTACCAGATTCA	qRT-PCR
Hs E2F4 Reverse	GCTCCATGCCTCCTTGTTCA	qRT-PCR
Hs HBB Forward	ACAACTGTGTTCACTAGCAACC	qRT-PCR
Hs HBB Reverse	GTTGCCCATAACAGCATCAGG	qRT-PCR
Hs HBG1/HBG2 Forward	AGGTGCTGACTTCCTTGGG	qRT-PCR
Hs HBG1/HBG2 Reverse	GGGTGAATTCTTTGCCGAA	qRT-PCR
Hs KLF1 Forward	AACACGGACCCATAGACAGC	qRT-PCR
Hs KLF1 Reverse	TTGCACGACAGTTTGGACAT	qRT-PCR
Hs GAPDH Forward	GCCAAAAGGGTCATCATCTC	qRT-PCR
Hs GAPDH Reverse	GGTGCTAAGCAGTTGGTGGT	qRT-PCR
Mm Hbb-b Forward	GCTGCATGTGGATCCTGAGA	qRT-PCR
Mm Hbb-b Reverse	CTTCTGGAAGGCAGCCTGTG	qRT-PCR
Mm Hbb-bh1 Forward	TGGGCTTGGGGGTTAAGAAC	qRT-PCR
Mm Hbb-bh1 Reverse	AACATGTTGCCCAGGAGCTT	qRT-PCR
Mm Hbb-y Forward	TTGGCTAGTCACTTCGGCAAT	qRT-PCR
Mm Hbb-y Reverse	GCATAGCGGACACACAGGAT	qRT-PCR
Mm Hba-a Forward	GCTGAAGCCCTGGAAAGGAT	qRT-PCR
Mm Hba-a Reverse	CAGAGCCGTGGCTTACATCA	qRT-PCR
Mm Hba-x Forward	CCGGTCAACTTCAAGCTCCT	qRT-PCR
Mm Hba-x Reverse	TGAACTTGTCCCAGGCTTCG	qRT-PCR
Mm Actb Forward	ACAGCAGTTGGTTGGAGCAAA	qRT-PCR
Mm Actb Reverse	GGGTGAGGGACTTCCTGTAA	qRT-PCR
Mm Gapdh Forward	TGGTGAAGCAGGCATCTGAG	qRT-PCR
Mm Gapdh Reverse	TGAAGTCGCAGGAGACAACC	qRT-PCR

Supplementary Table 1: List of shRNA clones and primer sets used.
